# A feasibility study of conducting surveillance for swine pathogens in slurry from North Carolina swine farms

**DOI:** 10.1038/s41598-020-67313-x

**Published:** 2020-06-22

**Authors:** Emily S. Bailey, Laura K. Borkenhagen, Jessica Y. Choi, Annette E. Greer, Marie R. Culhane, Gregory C. Gray

**Affiliations:** 1 Duke Global Health Institute, Duke University, Durham, NC USA; 2 Division of Infectious Diseases, Duke University School of Medicine, Durham, NC USA; 3 Julia Jones Matthews Department of Public Health, Graduate School of Biomedical Sciences, Texas Tech University Health Sciences Center, 1650 Pine Street, Abilene, TX 79601 USA; 4 Brody School of Medicine Department of Bioethics and Interdisciplinary Studies, East Carolina University, North Carolina Agromedicine Institute, Greenville, NC USA; 5 Department of Veterinary Population Medicine, College of Veterinary Medicine, University of Minnesota, St. Paul, MN USA; 6 Global Health Research Center, Duke-Kunshan University, Kunshan, China; 7 Emerging Infectious Diseases Program, Duke-NUS Medical School, Singapore, Singapore

**Keywords:** Environmental sciences, Risk factors, Environmental microbiology

## Abstract

Despite close contact between humans and animals on large scale farms, little to no infectious disease research is conducted at this interface. Our goal in this preliminary study was to explore if we could detect swine pathogens using a non-invasive, indirect approach through the study of swine slurry. From April to November 2018, 105 swine slurry samples were collected by farm personnel from waste pits at two sites on a swine farm in North Carolina. These samples were tested for DNA and RNA viruses using a real-time PCR and RT-PCR. Statistical analyses were performed to measure association between virus positive outcomes and potential predictors such as date of sample collection, weight of pigs, number of pigs in barn, temperature, and weather conditions. Overall, 86% of the samples had evidence of at least one of the targeted viruses. Ultimately, this study demonstrated the utility of conducting noninvasive surveillance for swine pathogens through the study of swine slurry. Such swine slurry surveillance may supplant the need to handle, restrain, and collect specimens directly from pigs thus providing an approach to emerging pathogen detection that appeals to the swine industry.

## Introduction

In farm environments, humans and animals are in frequent close contact where they are known to exchange zoonotic pathogens^1^. Despite this knowledge, sparse pathogen surveillance is conducted at this human–animal interface. Instead, emerging zoonotic pathogens are usually only detected when a pathogen’s impact is severe in either the animals or the animal workers that clinical investigations are subsequently sought. This results in missed opportunities for early detection and mitigation efforts.

Finding ways to collaborate with animal production industries is key to conducting such human-animal interface surveillance. Major industry objections that must be overcome include the biosecurity risks of permitting researchers to enter farms and the harm that the specimen collection may cause the animals. As there is increasing evidence that zoonotic viruses may be transmitted via environmental pathways such as through aerosol, feces, and water^2–7^, in this preliminary study we sought to engage swine farmers in periodically collecting fecal slurry samples from swine farms and to evaluate those samples for molecular evidence of zoonotic swine pathogens^8–10^. In this study the zoonotic pathogens are those that have the potential to pass between swine and humans, including influenza viruses, enteroviruses, coronaviruses, adenoviruses, and encephalomyocarditis virus. Such indirect and noninvasive fecal slurry surveillance reduces both the threats of biosecurity breaches and potential harm rendered in sampling production animals. These methods provided collection consistency and proved that established trustworthy partnerships of research engagement hold hope for future expansion of One Health research models. Our overall goal was to determine if our slurry sampling might be an acceptable method of conducting pathogen surveillance at the human-animal interface and yield robust calculations of prevalence of detected pathogens.

## Results

From the months of April through November 2018, a total of 105 swine slurry samples were collected from two sites on a farm in Eastern North Carolina. Overall, 90 (86%) of the 105 total swine slurry samples had evidence of at least one zoonotic virus. Four samples were positive for porcine reproductive and respiratory syndrome virus (PPRSv) (4%), one was positive for encephalomyocarditis virus (EMCV) (1%), three were positive for porcine circovirus 2 (PCV2) and PCV3 (3% respectively), and 20 samples (19%) were positive for senecavirus. 48 (46%) slurry samples were positive for adenovirus, 62 (59%) were positive for enterovirus, 39 (37%) samples were positive for coronavirus, and no samples were positive for influenza A, B, C, or D. 45 (94%) of 48 adenovirus positive specimens were successfully sequenced using partial genome sequencing and found to have clonal evidence of porcine adenovirus 5 (NCBI accession number AF289262.1). 42 (68%) of the 62 enterovirus positive specimens were successfully sequenced and were found to be a variety of human and porcine enteroviruses (Table 1), including porcine enteroviruses, human coxsackieviruses and echoviruses.

**Table 1:** Molecular subtyping results for enterovirus positive swine slurry samples.

Sample ID	Date collected	Enterovirus type	Accession number from NCBI GenBank
2	4/24/2018	Porcine enterovirus B	GQ502354.1
4	4/26/2018	Porcine enterovirus B	AM261011.1
5	5/1/2018	Enterovirus A NIE2014	KT717068.1
6	5/1/2018	Enterovirus G	KT265893.2
11	5/9/2018	Enterovirus G	KT265893.2
12	5/9/2018	Enterovirus G	KF705669.1
20	5/24/2018	Porcine enterovirus B	GQ502354.1
45	7/18/2018	Enterovirus G	MF113342.1
48	7/25/2018	Porcine enterovirus B	AM261011.1
49	7/25/2018	Coxsackievirus A4	KX021215.1
53	8/1/2018	Porcine enterovirus B	AM261020.1
61	8/14/2018	Enterovirus G	KF705660.1
62	8/16/2018	Enterovirus G	KY761948.1
66	8/23/2018	Porcine enterovirus B	GQ502354.1
69	8/30/2018	Porcine enterovirus B	AM261011.1
70	9/4/2018	Enterovirus A NIE2014	KT717068.1
71	9/4/2018	Enterovirus G	KT265893.2
73	8/27/2018	Enterovirus G	KT265893.2
76	9/16/2018	Enterovirus G	KT265946.1
77	9/16/2018	Human echovirus 11	JQ654098.1
78	9/18/2018	Enterovirus G	KT265973.1
79	9/18/2018	Echovirus E27	KC787137.1
80	9/6/2018	Echovirus JAA-2013	KC787146.2
83	9/25/2018	Enterovirus G	LC316821.1
85	9/27/2018	Enterovirus G	KY761948.1
86	10/2/2018	Coxsackievirus A10	KP164191.1
87	10/2/2018	Porcine enterovirus 10	JX219532.1
88	10/3/2018	Coxsackievirus A10	KP164191.1
89	10/3/2018	Enterovirus G	KT265882.1
91	10/9/2018	Porcine enterovirus 10	JX219532.1
92	10/11/2018	Coxsackievirus A10	KP164191.1
93	10/11/2018	Enterovirus G	KT265893.2
95	10/16/2018	Enterovirus G	KT265910.1
96	10/18/2018	Enterovirus G	KT265961.2
97	10/18/2018	Coxsackievirus B1	KU560979.1
99	10/27/2018	Enterovirus G	KT265961.2
100	11/1/2018	Enterovirus G	KP982873.1
101	11/1/2018	Porcine enterovirus 10	JX219532.1
102	11/7/2018	Enterovirus G	KP982873.1
103	11/7/2018	Porcine enterovirus 10	JX219532.1
104	11/8/2018	Coxsackievirus A10	KP164191.1

Enterovirus was the most prevalent virus with the greatest number of detections made during the months August through November. There were also multiple detections of adenovirus, coronavirus, and senecavirus. There were few detections of PRRSv, PCV2, PCV3, and EMCV. Bivariate risk factor studies were conducted for adenovirus, enterovirus, coronavirus, senecavirus, and any viral detection (Table 2). Adenovirus positivity was sometimes related to lower temperature and coronavirus positivity was related to extreme weather. Detections of both enterovirus and coronavirus were found to be associated with pig weight and the number of pigs in the barn, with more observed positives among the lowest three weight quartiles (45–295 lbs). Detections of adenovirus were found to be associated with the lowest weight quartile. Finally, detection of coronavirus and senecavirus often coincided with detection of enterovirus (OR 3.8; 95% CI 1.6, 9.2 and OR 3.6; 95% CI 1.1, 11.5, respectively).

**Table 2:** Unadjusted odds ratios for risk factors associated with virus positivity among 105 swine slurry samples.

Predictor	Adenovirus		Enterovirus		Coronavirus		Senecavirus		Any positives^a^	
	No. (%)	OR (95% CI)	No. (%)	OR (95% CI)	No. (%)	OR (95% CI)	No. (%)	OR (95% CI)	No. (%)	OR (95% CI)
**Month**										
July	1 (8.3)	0.10 (0.01, 1.02)	5 (41.7)	4.64 (0.71, 30.42)	3 (25.0)	1.33 (0.22, 8.22)	0 (0.0)	–	8 (66.7)	1.00 (0.20, 5.00)
August	6 (30.0)	0.50 (0.12, 1.97)	10 (50.0)	**6.50 (1.16, 36.57)**	6 (30.0)	1.71 (0.35, 8.37)	4 (20.0)	0.46 (0.11, 1.94)	8 (66.7)	2.83 (0.55, 14.47)
September	6 (42.9)	0.86 (0.20, 3.71)	13 (92.9)	**84.50 (6.80, 1,050.80)**	5 (35.7)	2.22 (0.42, 11.83)	9 (64.3)	3.34 (0.80, 13.94)	17 (85.0)	–
October	8 (57.1)	1.52 (0.35, 6.60)	13 (92.9)	**84.50 (6.80, 1,050.80)**	13 (92.9)	52.00 (0.474, 570.53)	0 (0.0)	–	14 (100.0)	–
November	3 (50.0)	1.14 (0.17, 7.60)	5 (83.3)	**32.50 (2.38, 443.14)**	6 (100.0)	–	0 (0.0)	–	6 (100.0)	–
April	4 (100.0)	–	4 (100.0)	–	0 (0.0)	–	0 (0.0)	–	4 (100.0)	–
May	12 (60.0)	1.71 (0.44, 6.63)	9 (45.0)	5.32 (0.94, 29.99)	3 (15.0)	0.71 (0.12, 4.11)	7 (35.0)	Ref	15 (75.0)	1.50 (0.34, 5.56)
June	7 (46.7)	Ref	2 (13.3)	Ref	3 (20.0)	Ref	0 (0.0)	–	10 (66.7)	Ref
**Weather**										
Sun	29 (44.6)	1.29 (0.38, 4.36)	38 (58.5)	1.64 (0.50, 5.43)	24 (36.9)	3.22 (0.66, 15.77)	9 (13.8)	0.88 (0.17, 4.66)	53 (81.5)	1.32 (0.32, 5.56)
Sun & wind	3 (37.5)	0.96 (0.16, 5.90)	5 (62.5)	1.94 (0.32, 11.76)	6 (75.0)	**16.50 (1.83, 148.61)**	4 (50.0)	5.50 (0.71, 42.60)	7 (87.5)	2.10 (0.18, 24.60)
Cloudy/overcast	8 (66.7)	3.20 (0.62, 16.49)	6 (50.0)	1.17 (0.24, 5.62)	5 (41.7)	3.93 (0.59, 26.11)	2 (16.7)	1.10 (0.13, 9.34)	12 (100.0)	–
Rain & wind	2 (28.6)	0.64 (0.09, 4.66)	6 (85.7)	1.94 (0.32, 11.71)	2 (28.6)	2.20 (0.24, 20.40)	3 (42.9)	4.12 (0.49, 34.49)	6 (85.7)	1.80 (0.15, 21.40)
Rain	5 (38.5)	Ref	6 (46.2)	Ref	2 (15.4)	Ref	2 (15.4)	Ref	10 (76.9)	Ref
**Temperature (°F)**										
< 70	6 (75.0)	**8.00 (1.25, 51.14)**	6 (75.0)	3.60 (0.59, 21.93)	4 (50.0)	2.14 (0.41, 11.17)	1 (12.5)	0.49 (0.05, 4.94)	8 (100.0)	–
70–79	4 (33.3)	1.33 (0.29, 6.12)	10 (83.3)	6.00 (1.06, 34.00)	7 (58.3)	3.00 (0.70, 12.88)	2 (16.7)	0.68 (0.11, 4.18)	10 (83.3)	1.11 (0.17, 7.17)
80–89	31 (49.2)	2.58 (0.89, 7.46)	35 (55.6)	1.50 (0.57, 3.98)	21 (33.3)	1.07 (0.38, 3.03)	12 (19.0)	0.80 (0.25, 2.60)	52 (82.5)	1.05 (0.30, 3.72)
90 +	6 (27.3)	Ref	10 (45.4)	Ref	7 (31.8)	Ref	5 (22.7)	Ref	18 (81.8)	Ref
**Pigs weight (lbs)**										
45–95 (Q1)	5 (16.1)	**0.18 (0.05, 0.61)**	14 (45.2)	2.12 (0.69, 6.51)	9 (29.0)	3.00 (0.72, 12.59)	6 (19.4)	1.76 (0.39, 7.89)	25 (80.6)	2.34 (0.70, 7.85)
100–200 (Q2)	14 (53.8)	1.08 (0.36, 3.24)	21 (80.8)	**10.80 (2.92, 39.99)**	12 (46.2)	**6.29 (1.50, 26.31)**	8 (30.8)	3.26 (0.75, 14.12)	25 (96.2)	**14.06 (1.62, 121.84)**
220–295 (Q3)	15 (65.2)	1.73 (0.54, 5.54)	19 (82.6)	**12.21 (3.05, 48.91)**	15 (65.2)	**13.75 (3.13, 60.42)**	3 (13.0)	1.10 (0.20, 6.09)	22 (95.6)	**12.38 (1.42, 107.74)**
300 (Q4)	13 (52.0)	Ref	7 (28.0)	Ref	3 (12.0)	Ref	3 (12.0)	Ref	16 (64.0)	Ref
**Number of pigs**										
1,000 +	22 (53.7)	1.81 (0.82, 3.99)	32 (78.0)	**4.29 (1.77, 10.43)**	16 (39.0)	1.14 (0.51, 2.56)	9 (22.0)	1.36 (0.51, 3.63)	35 (85.4)	1.21 (0.41, 3.57)
< 1,000	25 (39.1)	Ref	29 (45.3)	Ref	23 (35.9)	Ref	11 (17.2)	Ref	53 (82.8)	Ref
**Adenovirus**										
Positive	–	–	32 (68.1)	2.13 (0.96, 4.75)	18 (38.3)	1.09 (0.49, 2.42)	9 (19.2)	1.01 (0.38, 2.69)	–	–
Negative	–	–	29 (50.0)	Ref	21 (36.2)	Ref	11 (19.0)	Ref	–	–
**Enterovirus**										
Positive	32 (52.5)	2.13 (0.96, 4.75)	–	–	30 (49.2)	**3.76 (1.55, 9.15)**	16 (26.2)	**3.56 (1.10, 11.52)**	–	–
Negative	15 (34.1)	Ref	–	–	9 (20.4)	Ref	4 (9.1)	Ref	–	–
**Coronavirus**										
Positive	18 (46.2)	1.09 (0.49, 2.42)	30 (76.9)	**3.76 (1.55, 9.15)**	–	–	6 (15.4)	0.68 (0.24, 1.93)	–	–
Negative	29 (43.9)	Ref	31 (47.0)	Ref	–	–	14 (21.2)	Ref	–	–
**Senecavirus**										
Positive	9 (45.0)	1.01 (0.38, 2.69)	16 (80.0)	**3.56 (1.10, 11.52)**	6 (30.0)	0.68 (0.23, 1.93)	–	–	–	–
Negative	38 (44.7)	Ref	45 (52.9)	Ref	33 (38.8)	Ref	–	–	–	–

Bold text represents significant results.

Samples were collected from swine waste pits at two pig farms in North Carolina between April and November 2018.

*No.* number of positives, *OR* unadjusted odds ratio, *CI* confidence interval, *Ref.* referent group, *Q* quartile.

^a^Any positives outcome denotes one or more virus (adenovirus, enterovirus, coronavirus, encephalomyocarditis virus, porcine circovirus 2, porcine circovirus 3, porcine reproductive and respiratory syndrome virus, and senecavirus) detected in sample.

## Discussion

In this preliminary study, we conducted molecular surveillance for viruses in swine slurry samples collected from farms in Eastern North Carolina. Our overall goal was to determine if non-invasive slurry sampling was useful in the farm setting in detecting swine pathogens and supported by the farm owners. As such, the farmer was able to collect, freeze, and ship samples to our laboratory in Durham, North Carolina. Our unique approach of engaging farmers in developing research questions and including them in the presentation of results is representative of a shift in research focusing on animal production. This type of approach, better termed the One Health approach, brings together researchers across disciplines for the improvement of human, animal, and environmental health. The research presented here demonstrated that farmers are interested and willing to participate in research, so much so, that the farmer was willing to collect additional data on weather, number of pigs in the barn, etc. Although research working with farmers should consider specific factors such as farmer training, biosecurity, sample processing, and result dissemination, the relationships that come from working together to determine what questions are important to individuals growing food production animals are valuable for both parties and for public health as a whole.

Through our surveillance, we were able to identify the presence of multiple zoonotic viruses in slurry samples, such as coronaviruses and enteroviruses. Although, it is likely that influenza A virus was circulating within the swine population on the farm^3^, we were not able to identify this virus in the waste samples. Anderson et al., 2018 identified low concentrations of influenza A in swine slurry samples from farms in China; however, efforts to culture these viruses were not successful^3^. Similar to the results of our study, the largest detections of influenza in the Anderson et al., 2018 study were detected when the numbers of pigs on the farm were the greatest. In contrast to our results, Anderson et al., 2018 found that weather and temperature patterns were significantly related to positive detections of influenza A.

We did find considerable molecular evidence of adenovirus, enterovirus, and coronavirus using our pan-species molecular detection methods. These results are similar to other studies that have examined the species diversity of microorganisms in both swine and human waste^11^. Of particular interest is the detection of both animal and human enteroviruses (Table 1). We posit that these viruses were either present in water or food products given to the pigs or that there somehow was viral transmission between swine workers and the swine herd^12^.

Our analysis of temperature and weather indicates that in general these predictors were associated with increased adenovirus and coronavirus detections but that swine weight and the number of pigs in the barn were suggestive of increased enterovirus and coronavirus viral RNA positivity.

This research was limited by the lack of virus isolation data. As this was a preliminary study, virus isolation was not our goal. Samples have been archived for further characterization including infectivity experiments. Additionally, the statistical analysis was limited by the lack of a full year of data, which may have revealed additional viral associations with the seasonality of weather and temperature. Another limitation of this study is the lack of comparison to standard techniques used for swine pathogen sampling, a logical next step would be to perform a more complete comparison of this method to more traditional oral secretion detection methods.

Overall our findings demonstrated that zoonotic viruses (enteroviruses and coronaviruses) can be readily detected in swine slurry samples; as such, these samples may be used as an alternative non-invasive method for virus surveillance on swine farms. Future research directions should include a paired sample approach to link viral swine infections (through samples of swine nasal or oral secretions or feces) with swine waste.

## Materials and methods

### Site enrollment

A North Carolina, USA, swine farm with two barns was identified to participate in this preliminary study. Each barn had 12 pens with a center hallway and a fully slatted concrete floor over a deep pit to hold feces, urine, and waste water. Pits were emptied up to three times per year and recharged with recycled water. Farm personnel collected up to two slurry samples per week from the pit. A survey was completed for each sampling session and included information regarding date of collection, sampling site, sampling time, weight of pigs at site, number of pigs, and weather condition.

### Sample collection and processing

Slurry is defined as the feces and urine from pigs and the waste water used to remove the urine and feces from the pig pens^13^. Slurry samples from two swine barns containing finishing pigs were collected from approximately 5–10 cm below the surface of pits and were frozen at − 20 °C until shipped to our laboratory (a maximum of 24 h). Frozen samples and completed surveys were transported overnight to the Duke One Health Research Laboratory. Dates and pre-assigned sample numbers were used for sample tracking.

Slurry samples were diluted by methods previously described^14–16^. Briefly, samples were diluted at 10% w/v in sterile phosphate buffered saline (PBS) (pH 7.2). All samples were centrifuged for 1 min at 1,000*g* and 5 mL of the supernatant was centrifuged at 4 °C for 30 min at 1,500*g*. The remaining supernatant (~ 1.5 mL) was transferred to a sterile Eppendorf tube and centrifuged at 12,000*g* for 10 min. Finally, 1 mL of the supernatant was stored at − 80 °C until molecular testing was performed.

### Laboratory testing

We adapted previously published techniques for molecular evidence of both DNA and RNA viruses. Viral DNA was extracted from slurry samples using the QIAamp DNA Blood Mini Kit (QIAGEN, Inc., Valencia, CA) and tested with a real-time PCR (qPCR) assay for porcine circovirus 2 (PCV2)^17^ and porcine circovirus 3 (PCV3)^18^ using SsoAdvanced Universal Probes Supermix Real-Time PCR kit (BioRad, Inc., Hercules, CA). Viral DNA was also assessed using gel-based PCR assays with the Platinum Taq DNA Polymerase Kit (Thermo Fisher Scientific, Inc., Waltham, MA) for the detection of pan-species adenovirus^19^.

Viral RNA was extracted from slurry samples using the QIAamp Viral RNA Mini Kit (QIAGEN, Inc., Valencia, CA), and then assessed with real-time RT-PCR (qRT-PCR) assays using the SuperScript III Platinum One-Step qRT-PCR System with Platinum Taq DNA Polymerase (Thermo Fisher Scientific, Inc., Waltham, MA) for the detection of influenza A^20^, influenza B^21^, influenza C^22^, influenza D^23^, and encephalomyocarditis virus (EMCV)^24^. For the detection of porcine reproductive and respiratory syndrome virus (PRRSv), Tetracore EZ-PRRSv rRT-PCR assay was used (Tetracore, Inc., Rockville, MD). Additionally, viral RNA was assessed with gel-based RT-PCR assays using the SuperScript III Platinum One-Step RT-PCR System with Platinum Taq DNA Polymerase (Thermo Fisher Scientific, Inc., Waltham, MA) for the detection of pan-species enterovirus^25^, pan-species coronavirus (unpublished), and senecavirus^26^. Cell culture was not attempted for these specimens. Partial genome sequencing was performed by Eton Bioscience (Eton Bioscience, Inc., Raleigh, NC, USA) for positive specimens. Sequences were then compared to the NCBI sequence database using the BLAST application of BioEdit 7.1.9 (Ibis Biosciences, Carlsband, CA, USA). Sequences were aligned and phylogenetic analysis was performed using the UPGMA method in Geneious Prime 2019.1.1 (Biomatters Inc., San Diego, CA, USA).

### Statistical analysis

Chi-square test and Fisher’s exact test were performed to measure statistical association between potential predictors of the outcome of molecular assay positivity for each individual virus, as well as molecular evidence for any one or multiple viruses. Potential predictors included farm number, month of sample collection, time of sample collection, weight of pigs, number of pigs in barn, temperature, and weather conditions, as well as positivity for other viruses. Odds ratios and 95% confidence intervals were calculated for potential risk factors. Statistical analyses were performed using STATA 15.0 (StataCorp, College Station, TX).

### Ethics approval

This study was granted exemption from review status by the IACUC at Duke University on the grounds that the research did not include direct sample collection from animals.
